# Exploring the dynamic interactions between internet addiction, anxiety, and loneliness in adolescents: a longitudinal cross-lagged panel model

**DOI:** 10.3389/fpsyt.2025.1705792

**Published:** 2025-12-09

**Authors:** Yuntai Wang, Guodong Gong

**Affiliations:** Faculty of Education, Yunnan Normal University, Kunming, China

**Keywords:** adolescence, internet addiction, loneliness, anxiety, cross-laggedpanel model

## Abstract

**Purpose:**

The widespread adoption of the Internet has rendered adolescent Internet Addiction (IA) a major global public health concern, frequently co-occurring with psychological issues such as anxiety and loneliness. This study investigates their dynamic interrelationships.

**Methods:**

Using a longitudinal two-wave design, data were collected from 1,720 secondary school students in Yunnan Province, China, in October 2024 (T1) and March 2025 (T2). Measures included the Internet Addiction Test (IAT), the Generalized Anxiety Disorder Scale (GAD-7), and the UCLA Loneliness Scale (ULS). A cross-lagged panel model (CLPM) was employed to examine the causal pathways among IA, anxiety, and loneliness.

**Results:**

IA demonstrated significant temporal stability and positively predicted subsequent increases in both anxiety and loneliness. Furthermore, loneliness significantly predicted later levels of anxiety.

**Discussion:**

These findings extend the theoretical framework of the Compensatory Internet Use Model, providing empirical evidence for the dynamic mechanisms underlying adolescent mental health. The results underscore IA and loneliness as critical intervention targets, offering significant implications for clinical practice and educational policy aimed at Chinese adolescents.

## 1 Introduction

With the continuous rise in global Internet penetration, adolescent Internet addiction (IA) has emerged as a serious global public health concern. In Chinese mainland, approximately 10.32% adolescents are estimated to exhibit problematic internet use based on screening criteria (1). IA is characterized by excessive and uncontrolled Internet use that leads to distress or maladaptive functioning (2). Research indicates that adolescent IA is closely linked to various mental health issues, which not only heighten its risk but also exacerbate its negative outcomes, thereby creating a vicious cycle (3, 4).

Numerous studies have established that loneliness constitutes a significant risk factor for adolescent IA (5–7). Conceptually, loneliness is defined as the discomfort experienced when anticipated social interactions differ from those actually realized (8). A variety of investigations have explored the interplay between loneliness and IA, revealing a moderate positive correlation between the two (5). Additionally, research focusing on Chinese adolescents indicates that loneliness mediates the relationship between childhood trauma and IA, underscoring its role as a psychological pathway to IA (9). Furthermore, studies have demonstrated that loneliness-related avoidance behaviors are strong predictors of adolescent IA, with these tendencies potentially exacerbating Internet dependence, particularly among introverted or shy adolescents (10). Importantly, anxiety has been identified as a crucial factor influencing the relationship between loneliness and IA (11).

Anxiety disorders are defined as psychiatric conditions characterized by excessive and persistent worry and fear. Studies have demonstrated a significant correlation between IA and the prevalence of anxiety disorders. Adolescents suffering from anxiety disorders are more likely to seek emotional support online, a behavior that can further exacerbate IA (12). Moreover, the relationship between anxiety disorders and IA is bidirectional: anxiety can drive excessive Internet use, while excessive use may, in turn, intensify anxiety symptoms (11). The interplay among anxiety, loneliness, and IA is notably complex. Evidence suggests that loneliness may mediate the association between IA and anxiety, with both anxiety and loneliness significantly increasing the subsequent risk of IA; furthermore, anxiety can indirectly affect IA through loneliness (13). Additionally, research indicates that anxiety may trigger loneliness among adolescents, leading them to rely on the Internet for emotional support or social engagement, which ultimately heightens the risk of IA (14).

The close comorbidity among IA, loneliness, and anxiety can be theoretically explained through multiple pathways. The Model of Compensatory Internet Use suggests that IA often develops when individuals resort to online interactions in the virtual world to alleviate negative emotions and difficulties related to loneliness in real life (15). To be more specific, when individuals experience negative states in reality—such as loneliness, anxiety, stress, or lack of social skills—they may turn to the online world to seek fulfillment or relief that is otherwise unavailable offline. (16, 17). Initially, these online activities (e.g., social media use, online gaming, information browsing) may serve as an adaptive coping strategy by offering temporary alleviation of negative emotions. However, the model further posits that if this compensatory use becomes excessive, rigid, and uncontrolled, it shifts from being adaptive to maladaptive. The anticipated negative outcome is the development of IA. This is because over-reliance on online compensation can undermine an individual’s motivation and capacity to resolve problems in the real world, potentially leading to further withdrawal from offline social interactions, deterioration of real-life relationships, and impaired emotion regulation skills. Consequently, a vicious cycle is formed: offline problems drive online compensation, excessive compensation exacerbates IA, and addictive behaviors, in turn, intensify psychological distress (e.g., anxiety and loneliness) in reality. (17).

Although the associations among IA, loneliness, and anxiety have been well established, critical knowledge gaps persist regarding their dynamic and time-evolving causal relationships. Most of the evidence comes from cross-sectional studies, a design inherently incapable of elucidating the direction of mutual influence between variables or determining the existence of more complex bidirectional or multidirectional reciprocal relationships. Secondly, while some longitudinal studies have emerged recently, the majority have focused exclusively on dyadic relationships between pairs of variables (e.g., examining only IA and anxiety, or loneliness and IA). (e.g., 7, 18, 19). Therefore, while the impact of IA on mental health has been extensively studied, its bidirectional dynamic relationships with anxiety and loneliness remain underexplored. The scarcity of longitudinal evidence, in particular, constrains professionals’ ability to address emerging challenges, such as the surge in Internet dependence in the post-pandemic era (20), thereby hindering the development of effective and targeted intervention strategies.

Based on this, this study employs a Cross-Lagged Panel Model (CLPM) to analyze two-wave longitudinal data from adolescents, with the objective of investigating the time-varying interactions and causal linkages among IA, anxiety, and loneliness. Specifically, the study addresses two key questions: (1) Does IA at Time 1 significantly predict levels of anxiety and loneliness at Time 2? (2) Does loneliness at Time 1 significantly predict IA at Time 2? By conducting a detailed analysis of the cross-lagged effects, this research aims to transcend simple correlational descriptions and provide robust longitudinal evidence for understanding the mechanisms of comorbidity and the vicious cycles underlying these prevalent mental health issues. The findings will yield evidence-based implications for clinical interventions and preventive strategies, thereby contributing to global efforts in addressing mental health challenges.

## 2 Method

### 2.1 Participants

Data were collected using a convenience sampling method from junior and senior high school students at a public secondary school in Yunnan Province, China. The data collection occurred in two phases: the first in October 2024 and the second in March 2025. The inclusion criteria for this study were: (1) enrolled students at the public secondary school during the survey period; (2) aged between 12 and 18 years; (3) provided informed assent (from themselves) and consent (from their guardians). Exclusion criteria included: (1) students who were absent (e.g., due to leave) and failed to participate in both waves of data collection; (2) students with diagnosed severe cognitive impairments or psychiatric disorders (as per school records and homeroom teacher confirmation) that prevented them from completing the questionnaires. Ultimately, all students who completed both surveys and provided valid data were included in the analysis.

In each phase, data collection was conducted simultaneously by classroom, with two master’s students in applied psychology administering the surveys in each class. All research assistants received uniform training in standardized administration procedures prior to data collection. Participants completed the assessments using offline paper-and-pencil questionnaires and reported their age and gender, with gender coded as 1 for male and 2 for female.

After merging the two waves of data based on student identification numbers, a total of 1,720 students provided valid data regarding IA, loneliness, and anxiety, resulting in an effective response rate of 91.05%. Informed consent was obtained from all participants and their guardians prior to participation. The consent form clearly articulated the study’s objectives, assured the anonymity of all data, and emphasized that participation was entirely voluntary, with the right to withdraw at any time without penalty. Approval for the survey was granted by the local education bureau and the school principal. All study materials and procedures were thoroughly reviewed and approved by the Scientific Review Group of the Applied Psychology Program, Faculty of Education, Yunnan Normal University (IRB No. 2024005).

### 2.2 Measures

#### 2.2.1 Internet addiction test

Kimberly Young’s Internet Addiction Test (IAT) is one of the most widely utilized diagnostic tools for assessing IA (21). The Chinese version of the IAT has exhibited strong reliability and validity among Chinese adolescents, we adopted the cutoff score proposed by Lai et al. (22), using a total IAT score of ≥ 50 to indicate the presence of IA problems (22). Each item was evaluated using a 5-point Likert scale ranging from 1 to 5 and the total score was treated as a continuous variable in all analyses (23). In the present study, the internal consistency of the scale was satisfactory, with a Cronbach’s α of 0.92 at both T1 and T2.

#### 2.2.2 General anxiety disorder-7

The Chinese version of the 7-item Generalized Anxiety Disorder scale (GAD-7; 24) was utilized to assess anxiety. Items are rated on a 4-point scale, ranging from 0 (not at all) to 3 (nearly every day). The total score ranges from 0 to 21, with the commonly used severity cut-offs as follows: 0-4 = minimal anxiety, 5-9 = mild anxiety, 10-14 = moderate anxiety, and 15-21 = severe anxiety. The GAD-7 has demonstrated strong reliability among Chinese adolescents (25). In the present study, the internal consistency was found to be adequate, with Cronbach’s α values of 0.92 at T1 and 0.89 at T2.

#### 2.2.3 UCLA loneliness scale

Loneliness was assessed utilizing the UCLA Loneliness Scale (26), which comprises 20 items rated on a 4-point scale ranging from 1 (never) to 4 (always). The UCLA Loneliness Scale has demonstrated strong reliability among Chinese adolescents (27). In the current study, the scale exhibited satisfactory internal consistency, with a Cronbach’s α of 0.93 at T1 and 0.92 at T2.

### 2.3 Data analysis

#### 2.3.1 Common method bias

Given that all data were assessed through self-report measures, Harman’s single-factor test was employed to detect potential common method bias across the two time points (28). The analyses were conducted using IBM SPSS Statistics version 27.0 Prior research indicates that serious common method bias is absent when the first factor accounts for less than 40% of the total variance. In alignment with this prior research, common method bias was deemed negligible in this study as the variance explained by the first factor was below 40%, suggesting no significant threat of common method bias (CMB) (28).

#### 2.3.2 Longitudinal measurement invariance

Given that data were collected at two time points, a multiple-group confirmatory factor analysis (CFA) was conducted using Mplus 8.3 to test the longitudinal measurement invariance of IA, anxiety, and loneliness scores. This analysis ensured that the constructs retained consistent meanings across the two time points (29). The anxiety scale had a limited number of items, with each item treated as an individual indicator. In contrast, the loneliness and IA scales contained a greater number of items, which could significantly increase model complexity and lead to the underestimation of model fit indices (30). To mitigate this issue, items from the loneliness and IA scales were parceled according to their respective scale dimensions (30). The assessment of measurement invariance was based on the absolute changes in the Comparative Fit Index (ΔCFI) and Tucker–Lewis Index (ΔTLI), as well as changes in the Root Mean Square Error of Approximation (ΔRMSEA). Measurement invariance was considered supported when the absolute values of ΔCFI ≤ 0.01, ΔTLI ≤ 0.01, and ΔRMSEA ≤ 0.015 (30, 31).

#### 2.3.3 Cross-lagged analysis

The CLPM examining the relationships among IA loneliness, and anxiety was constructed using Mplus version 8.3. All models were estimated utilizing robust maximum likelihood estimation (MLR). Model fit was assessed through the Comparative Fit Index (CFI), Tucker–Lewis Index (TLI), Root Mean Square Error of Approximation (RMSEA), and Standardized Root Mean Square Residual (SRMR). According to the guidelines established by Browne and Browne & Cudeck (32) and Hu & Bentler (33), CFI and TLI ≥ 0.90, RMSEA ≤ 0.08, and SRMR ≤ 0.08 indicate an acceptable model fit. Conversely, CFI and TLI ≥ 0.95, RMSEA ≤ 0.06, and SRMR ≤ 0.06, signify a good model fit. To enhance model simplicity and facilitate comparisons in model fit, chi-square difference tests (Δ*χ²*) were conducted. In cases where the chi-square difference between two models was non-significant, the more parsimonious model was preferred; conversely, if the difference was significant, the model accounting for greater variance was selected as the optimal model (34).

After establishing measurement invariance, the researchers specified a CLPM to examine the temporal associations among IA, loneliness, and anxiety. The model simultaneously evaluated three components: (1) cross-lagged paths between variables across time points, (2) synchronous correlations among variables measured at the same time point, and (3) correlated measurement errors for the same indicators over time. To simplify the model and enhance interpretability, a freely estimated baseline model (M1) was initially tested. This was followed by model trimming to develop a more parsimonious model (M2) (35). Guided by theoretical justification and the goal of optimizing model fit, additional paths were incorporated based on modification indices (MIs) and relevant theoretical recommendations, resulting in the final model (M3). For interpreting the magnitude of cross-lagged effects, the study adopted the criteria proposed by Orth et al. (36), where standardized cross-lagged coefficients of 0.03, 0.07, and 0.12 correspond to small, medium, and large effect sizes, respectively (36).

## 3 Results

### 3.1 Demographic characteristics

The gender distribution among participants was approximately balanced, with ages ranging from 11 to 20 years (*Mean* ± *SD* = 14.46 ± 1.55). The majority of participants were of Han ethnicity, came from families with multiple children, were born in urban areas, and had parents with stable marital statuses. Additional demographic information is provided in **Table 1**.

**Table 1:** Demographic Information (N = 1,720).

Variables	Frequency	Percentage
Gender		
Male	882	51.28%
Female	838	48.72%
Age		
Junior adolescence (Age 14 and under)	1054	61.28%
Senior adolescence (Age 15 and over)	666	38.72%
Ethnic		
Han	1476	85.81%
Minor	244	14.19%
Family Fertility		
Only-child families	406	23.60%
Multi-child families	1314	76.40%
Birthplace		
Urban	1578	91.74%
Rural	142	8.26%
Parents’ marriage		
Good	1594	92.67%
Divorced	126	7.33%

### 3.2 Common analysis bias

Harman’s single-factor test was performed to evaluate common method bias across two time points (28). The results revealed that seven factors had eigenvalues exceeding 1 at both time points. The first factor explained 30.58% of the variance at Time 1 and 30.46% at Time 2, both of which fall below the critical threshold of 40%. This indicates that common method bias was not a significant concern in this study (28).

### 3.3 Statistical description and correlation analysis

**Table 2** presents the means, standard deviations, and correlations for anxiety, IA loneliness, and demographic variables, specifically age and gender. Correlational analyses revealed significant positive cross-time associations for all three psychological variables. Specifically, anxiety at T1was positively correlated with anxiety at T2 (*r* = 0.64, *p* <.001), IA at T1 was positively correlated with IA at T2 (*r* = 0.68, *p* <.001), and loneliness at T1 was positively correlated with loneliness at T2 (*r* = 0.36, *p* <.001). These findings indicate strong temporal stability for both anxiety and IA, and moderate stability for loneliness across the two measurement points. The results demonstrated that anxiety, IA, and loneliness were significantly and positively correlated at both T1 and T2, which supports the validity of the cross-lagged analysis. Furthermore, age, ethnic, family fertility, birthplace, parents’ marriage and gender were significantly associated with anxiety, IA, and loneliness; thus, they were included as covariates in the cross-lagged analysis to statistically control for their effects.

**Table 2:** Statistical description and correlation analysis.

Variable	M	SD	1	2	3	4	5	6	7	8
1.Gender	1.49	0.50	—							
2.Age	14.11	1.56	0.02	—						
3.Anxiety T1	4.49	4.78	0.17**	0.04	—					
4.IA T1	45.63	15.31	0.05*	0.15***	0.51***	—				
5.Loneliness T1	48.67	6.27	0.16**	0.13***	0.38***	0.36***	—			
6.Anxiety T2	4.39	4.31	0.14**	0.09***	0.64***	0.42***	0.32***	—		
7.IA T2	44.52	14.76	0.04	0.28***	0.38***	0.68***	0.27***	0.48***	—	
8.Loneliness T2	47.74	5.66	0.16**	0.15***	0.21***	0.21***	0.36***	0.28***	0.32***	—

*p <.05, **p <.01, ***p <.001, two-tailed.

### 3.4 Longitudinal measurement invariance

**Table 3** presents the model fit results for the configural, metric, and scalar invariance models of the study variables. The results indicate that all models meet the established criteria for evaluating measurement invariance. Based on the chi-square difference tests and changes in fit indices (ΔCFI, ΔTLI, ΔRMSEA), anxiety and IA demonstrated scalar invariance, while loneliness achieved metric invariance. These findings support the examination of longitudinal relationships among the study variables.

**Table 3:** Measurement invariance test of variables.

Variable	*χ² (df)*	CFI	TLI	RMSEA	Δ*χ²* (Δ*df*)	ΔCFI	ΔTLI	ΔRMSEA
Anxiety								
M1	414.55 (76)	0.960	0.950	0.051				
M2	433.486 (82)	0.960	0.956	0.050	18.936 (6)	0.000	0.006	-0.001
M3	494.515 (89)	0.956	0.953	0.051	61.029 (7)	-0.004	-0.003	0.001
Internet addiction								
M1	346.078 (45)	0.973	0.961	0.062				
M2	396.483 (51)	0.969	0.960	0.063	50.405 (6)	-0.001	0.001	0.001
M3	878.371 (58)	0.927	0.917	0.079	481.888 (7)	-0.043	0.016	0.016
Loneliness								
M1	0.093 (1)	0.999	1.000	0.001				
M2	10.592 (2)	0.995	0.984	0.050	10.499 (1)	-0.004	-0.016	0.050
M3	337.019 (4)	0.792	0.688	0.220	326.427 (2)	-0.203	-0.296	0.170

M1: Configural invariance M2: Metric invariance M3: Scalar invariance.

### 3.5 Cross-lagged panel analysis

**Table 4** presents the fit indices and model comparison results for the CLPMs. Initially, all three variables were freely estimated across the two time points, with gender, age, family fertility, ethnic, birthplace, and parents’ marriage included as covariates. To identify the most parsimonious model guided by the principle of parsimony and theoretical tenets of cross-lagged modeling, we constrained all non-significant paths in the baseline model, resulting in Model M2. The constrained paths included Loneliness at T1predicting IA at T2, Anxiety at T1 predicting Loneliness at T2, Anxiety at T1 predicting IA at T2, and the effects of Gender on IA at T2 and Anxiety at T2.

**Table 4:** Model fit indices and model comparisons.

Model name	CFI	TLI	RMSEA	SRMR	*χ²*	*df*	ΔCFI	ΔRMSEA	Δ*χ²*	Δ*df*	*p*
M1	0.936	0.809	0.113	0.06	138.153	6					
M2	0.942	0.883	0.088	0.061	130.149	9	0.006	0.025	8.004	3	0.046
M3	0.998	0.99	0.026	0.01	6.425	3	0.001	0.008	123.724	6	0.000

**Figure 1:**
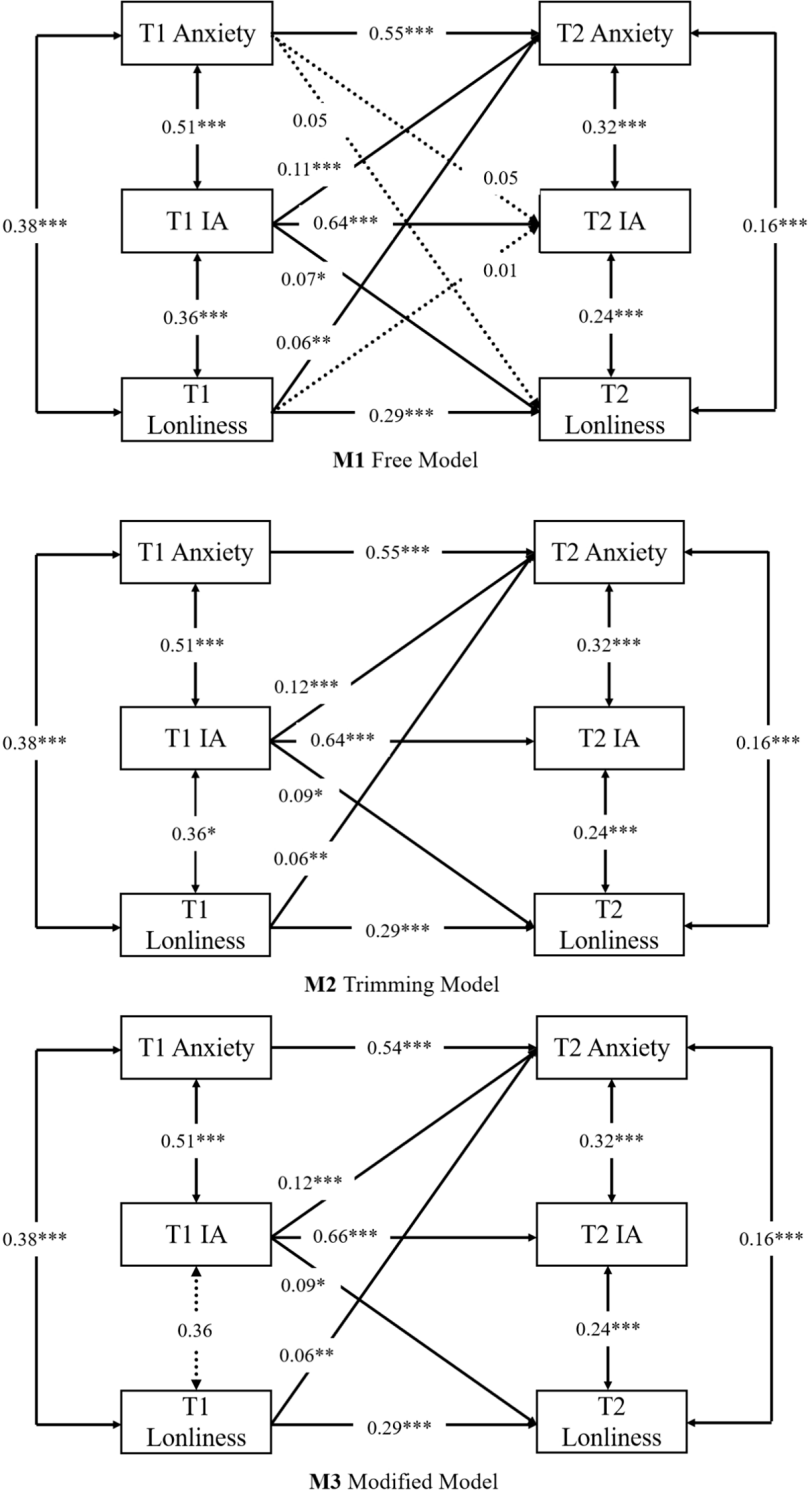
Cross-lagged panel analysis. *p < .05, **p < .01, ***p < .001.

Based on the modification indices (MIs) and the results of the correlation analyses, we subsequently incorporated synchronous correlations between gender and both anxiety and loneliness at T1. This modification resulted in a saturated model, which suggested potential overfitting. Consequently, we employed a stepwise trimming approach to eliminate non-significant paths, thereby enhancing model parsimony and fit. The final model, M3, exhibited an excellent fit to the data (CFI = 0.99 ≥ 0.90, TLI = 0.99 ≥ 0.90, RMSEA = 0.026 ≤ 0.08, SRMR = 0.01). The findings indicated that M3 provided a significantly improved fit compared to Models M1 and M2. Due to its simplicity and superior fit, M3 was chosen as the final model for further interpretation. **Figure 1** displays the specific paths for the three models.

**Figure 1** M3 illustrates the standardized results of the cross-lagged model after controlling for covariates such as age and gender, as well as autoregressive effects and synchronous correlations. The findings demonstrated a strong stability of anxiety, IA, and loneliness across the two measurement points, with autoregressive path coefficients of 0.54, 0.66, and 0.29, respectively, all of which were statistically significant (*p* <.001). Specifically, IA at T1 significantly and positively predicted anxiety at T2 (*β* = 0.12, 95% CI [0.07, 0.17], *p* <.001) and loneliness at T2 (*β* = 0.09, 95% CI [0.04, 0.14], *p* <.05), both exhibiting medium effect sizes. Additionally, loneliness at T1 significantly and positively predicted anxiety at T2 (*β* = 0.06, 95% CI [0.02, 0.07], *p* <.01), although the effect size was relatively small yet statistically significant.

## 4 Discussion

This study, utilizing two-wave longitudinal data, represented the inaugural effort to establish a CLPM examining the interrelations among IA, anxiety, and loneliness in adolescents. It elucidates their distinctive and dynamic bidirectional associations. By comparing three CLPMs, we investigated the potential mechanisms linking IA, anxiety, and loneliness, ultimately identifying the best-fitting model (M3). The findings can be summarized in two key points: First, IA, anxiety, and loneliness exhibited significant temporal stability. Second, IA significantly predicted subsequent anxiety and loneliness over time.

Specifically, our findings revealed that adolescent IA positively predicted subsequent anxiety symptoms, aligning with previous research and underscoring its role as a risk factor in the maintenance and escalation of anxiety (17). Prior studies have demonstrated a strong link between IA and deficits in executive functioning among adolescents, which may lead to a diminished capacity for cognitive and emotional regulation (37). Neurobiological evidence suggests that the prefrontal cortex—central to executive functioning—demonstrates markedly reduced inhibitory control in IA patients, potentially compromising their capacity to regulate online behaviors and subsequently intensifying anxiety (38). Additionally, adolescents frequently utilize the internet as a means of escape when confronted with real-life stressors, potentially undermining their ability to cope with real-world challenges and exacerbating anxiety symptoms (39). Our findings are consistent with the Model of Compensatory Internet Use. This model suggests that individuals may resort to the Internet as an alternative avenue for fulfilling unmet psychological needs in real life, such as alleviating negative emotions. Our results indicate that IA significantly predicts subsequent anxiety and loneliness, which aligns with the model’s proposition that compensatory use may lead to maladaptive outcomes (17, 40). Encouraging adolescents to engage in physical activity can enhance emotional regulation and directly alleviate anxiety symptoms (41). Furthermore, social support has been identified as a crucial protective factor that may mitigate the predictive effect of IA on anxiety (42).

IA is a significant risk factor for the maintenance and escalation of loneliness among adolescents, consistent with previous research findings (17, 43). Excessive use, a core symptom of IA (11), may diminish real-world social opportunities and impair social skills, thereby exacerbating feelings of loneliness (44). This phenomenon aligns with the concept of self-regulation failure within social cognitive theory, wherein individuals struggle to effectively manage their behaviors or emotions when confronted with external stimuli, leading to maladaptive consequences (45). In the context of the internet era, adolescents who fail to manage their online time effectively may gradually lose interest in real-world social interactions, ultimately fostering an excessive reliance on the virtual world (45). Evidence indicates that enhancing adolescents’ psychological needs—such as fostering a sense of belonging and autonomy in real life—may help mitigate internet dependency (40).

Interestingly, our findings did not support the predictions of the Model of Compensatory Internet Use, as neither anxiety nor loneliness emerged as significant predictors of IA. This finding offers an important nuance and extension to the Model of Compensatory Internet Use. First, it may relate to the failure of compensation and the ensuing vicious cycle. While individuals might initially turn to the Internet to compensate for anxiety or loneliness (15), once this use escalates into addiction, its nature changes. Excessive and uncontrolled Internet use is maintained through negative reinforcement—the behavior is repeated to relieve or escape aversive real-life states, but in doing so, it impairs real-world social opportunities, problem-solving skills, and emotion regulation capacities (17). Consequently, IA transitions from being a mere “consequence” to an active “engine” that exacerbates psychological problems in a vicious cycle. Second, differences in time windows could be a crucial factor. The progression from psychological distress to addictive behavior might be a more protracted, gradual process, the effects of which are not easily captured over a short four-month period. In contrast, the negative impact of addictive behavior on daily functioning and socio-emotional well-being might manifest more rapidly, making it more detectable within our study design.

Consistent with previous research, loneliness positively predicts individuals’ anxiety levels (46, 47). Individuals experiencing loneliness often lack effective emotion regulation strategies, which may exacerbate their anxiety symptoms (48). Furthermore, adolescents, being at a critical stage of social and emotional development, may experience heightened sensitivity and insecurity in social contexts, which can initiate or exacerbate anxiety symptoms (49, 50). Additionally, the co-occurrence of loneliness and anxiety may be closely tied to adolescents’ social needs and self-perceptions. Loneliness is often accompanied by negative evaluations of social relationships and doubts about self-worth, which can further intensify anxiety (50, 51). From a neurobiological perspective, the link between loneliness and anxiety may stem from changes in specific brain regions among adolescents. Studies have shown that loneliness significantly affects neuronal activity in the prefrontal cortex and amygdala, thereby intensifying anxiety symptoms in this population (52, 53). Enhancing adolescents’ social skills, providing supportive social networks, and improving self-perception can effectively mitigate loneliness and its adverse effects on anxiety (54).

In summary, this study is the first to utilize longitudinal data to construct a cross-lagged model, thereby demonstrating the potential causal predictive effects of IA on anxiety and loneliness. This research provides dynamic evidence for the comorbidity mechanisms associated with adolescent mental health. By employing a large and representative sample, this study effectively addresses the existing gap in empirical research concerning adolescent IA. The findings carry significant implications: First, IA and its related mental health issues in adolescents can serve as critical intervention targets in clinical practice. Second, the results offer evidence-based guidance for educational policies and social interventions, underscoring the importance of fostering real-world social skills in the digital era. Finally, this study theoretically extends the Model of Compensatory Internet Use. Overall, it deepens the understanding of the mechanisms underlying adolescent mental health issues and provides valuable insights for psychological screening, intervention strategies, and the development of social support systems.

## 5 Limitation and future research

Although this study innovatively employed longitudinal data to construct a CLPM examining the interrelationships between IA, loneliness, and anxiety among adolescents, thereby providing a dynamic perspective on the mechanisms of mental health comorbidity. However, several limitations persist that warrant attention in future research. Firstly, the relatively short time span, with only two waves of data collected four months apart, may not adequately capture the short-term dynamic changes between the variables and could overlook the predictive effects of anxiety and loneliness on IA. Secondly, the participants were exclusively recruited from public schools in Yunnan Province, China, without incorporating clinical or cross-cultural populations, which may limit the broader applicability and external validity of the study’s findings. Besides, the two-wave design of the present study precluded the application of the RI-CLPM. Future research employing three or more waves of data to utilize the RI-CLPM to reveal pure within-person dynamic processes represents a highly valuable direction. Lastly, although demographic covariates (gender, age, family fertility, ethnic, birthplace, and parents’ marriage) were controlled as confounding variables, the cross-lagged model may still be influenced by unmeasured confounders.

Building on the current findings, future research should extend the tracking period and increase the frequency of data collection to more accurately capture the dynamic changes between variables. Furthermore, it is essential to validate these results across diverse cultural contexts, such as by comparing adolescents in collectivist versus individualist societies. Advancing these research directions will help elucidate the complex causal relationship between IA and mental health, thereby providing a theoretical foundation for enhancing psychological well-being in the digital era.

## 6 Conclusion

This study, utilizing longitudinal data for the first time, employed a CLPM to demonstrated that adolescent IA significantly predicts both anxiety and loneliness, while loneliness also predicts anxiety. These findings extend the Model of Compensatory Internet Use, indicating that excessive Internet use exacerbates psychological distress rather than alleviating real-world difficulties. Additionally, this research provides dynamic causal evidence for comorbidity mechanisms in adolescent mental health, highlighting IA and related loneliness as critical targets for intervention. Our results address a significant empirical gap and offer a theoretical foundation for developing precise intervention strategies and social support systems in the digital era.

## Data Availability

The raw data supporting the conclusions of this article will be made available by the authors, without undue reservation.
